# Algorithms for Computing the Triplet Quartet Distances for Binary General Trees

**DOI:** 10.3390/biology2041189

**Published:** 2013-09-26

**Authors:** Andreas Sand, Morten K. Holt, Jens Johansen, Rolf Fagerberg, Gerth Stølting Brodal, Christian N. S. Pedersen, Thomas Mailund

**Affiliations:** 1 Department of Computer Science, Aarhus University, IT-Parken, Aabogade 34, DK-8200 Aarhus N, Denmark; E-Mails: asand@birc.au.dk (A.S.); the.thawk@gmail.com (M.K.H.); jens.joha@gmail.com (J.J.); gerth@cs.au.dk (G.S.B.); cstorm@birc.au.dk (C.N.S.P.); 2 Bioinformatics Research Centre, Aarhus University, C.F. Møllers Allé 8, DK-8000 Aarhus C, Denmark; 3 Department of Mathematics and Computer Science, University of Southern Denmark, Campusvej 55, DK-5230 Odense M, Denmark; E-Mail: rolf@imada.sdu.dk; 4 MADALGO -Center for Massive Data Algorithms, a Centre of the Danish National Research Foundation, Aabogade 34, DK-8200 Aarhus N, Denmark

**Keywords:** algorithmic development, tree comparison, triplet distance, quartet distance

## Abstract

Distance measures between trees are useful for comparing trees in a systematic manner, and several different distance measures have been proposed. The triplet and quartet distances, for rooted and unrooted trees, respectively, are defined as the number of subsets of three or four leaves, respectively, where the topologies of the induced subtrees differ. These distances can trivially be computed by explicitly enumerating all sets of three or four leaves and testing if the topologies are different, but this leads to time complexities at least of the order *n*^3^ or *n*^4^ just for enumerating the sets. The different topologies can be counted implicitly, however, and in this paper, we review a series of algorithmic improvements that have been used during the last decade to develop more efficient algorithms by exploiting two different strategies for this; one based on dynamic programming and another based on coloring leaves in one tree and updating a hierarchical decomposition of the other.

## 1. Introduction

Evolutionary relationships are often represented as trees; a practice not only used in biology, where trees can represent, for example, species relationships or gene relationships in a gene family, but also used in many other fields studying objects related in some evolutionary fashion. Examples include linguistics, where trees represent the evolution of related languages, or archeology, where trees have been used to represent how copies of ancient manuscripts have changed over time. Common for most such fields is that the true tree relationship between objects is never observed, but must be inferred, and depending on both the data available and the methods used for the inference, the inferred trees will likely be slightly different.

Tree distances provide a formal way of quantifying how similar two trees are and, for example, determine if two trees are significantly similar or no more similar than could be expected by chance. Many different distances have been defined on trees. Most only consider the tree topology, *i.e.*, how nodes are related, but not how branch lengths might differ between the trees, and most of these only consider relationships between labeled leaves and consider inner nodes as unlabeled. Commonly used distance measures are the Robinson-Foulds distance [1] and the quartet distance [2] for unrooted trees and the triplet distance [3] for rooted trees. All three are based on the idea of enumerating all substructures of the two trees’ topology and counting how often the structure is the same in the two trees and how often it is different.

The Robinson-Foulds distance considers all the ways the leaf labels can be split into two sets and counts how often only one of the trees has an edge matching this split. Informally, this essentially means that it counts how often the two trees have the “same edge” and how often an edge in one tree has no counterpart in the other. Edges are arguably the simplest element of the topology of a tree, and not surprisingly, the Robinson-Foulds distance is both the most frequently used distance measure and the distance measure that can be computed with the optimal algorithmic complexity, *O*(*n*), for trees with n leaves [4].

The triplet and quartet distances enumerate all subsets of three or four leaves, respectively, and count how often the topologies induced by the three or four leaves are the same in the two trees. The triplet distance is intended for rooted trees, where the triplet topology is the smallest informative subtree (for unrooted trees, all subtrees with three leaves have the same topology), while the quartet distance is intended for unrooted trees, where the quartet topology is the smallest informative subtree. Whether it is possible to compute the triplet and quartet distance in linear time is unknown. The fastest known algorithms have time complexity *O*(*n* log *n*) for both distances for binary trees, *O*(*n* log *n*) for the triplet distance and *O*(*dn* log *n*) for the quartet distance for general trees, where d is the largest degree of a node in the trees [5].

In this paper, we will review the algorithmic development that led to these non-trivial running times, in particular, the development of algorithms for general (non-binary) trees to which the authors have contributed a number of papers. We will first formally define the triplet and quartet distance between two leaf-labeled trees. We then describe the state-of-the-art for binary trees with two different approaches to computing the distances: one based on dynamic programming with time complexity *O*(*n*^2^) for both quartet and triplet distance and one based on coloring leaves in a tree traversal with complexity *O*(*n* log *n*) for quartet distances (with very little work, this algorithm can be modified to compute the triplet distance, but it has never been described as such in the literature). The dynamic programming approach was the first algorithm that was modified to deal with the quartet distance for general trees, and in the following section, we describe a number of approaches to doing this. In the same section, we describe how the tree coloring approach can be adapted to general trees, leading to the currently best worst-case running times mentioned above. We then turn to practical implementations, present experimental results for the best algorithms and show that the theoretically fastest algorithms can also be implemented to run efficiently in practice.

## 2. The Triplet and Quartet Distances

Given two trees, *T*_1_ and *T*_2_, each with *n* leaves with labels from the same set of *n* labels, the triplet distance is defined for rooted trees, while the quartet distance is defined for unrooted trees.

**Figure 1:**
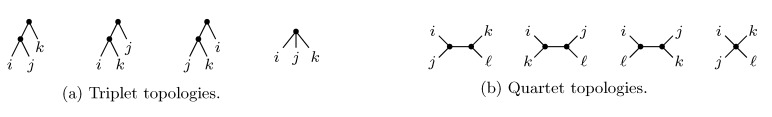
Different cases for triplet and quartet topologies.

A triplet is a set, {*i*, *j*, *k*}, of three leaf labels. This is the smallest number of leaves for which the subtree induced by these leaves can have different topologies in two rooted trees. The possible topological relationships between leaves *i*, *j* and *k* are shown in Figure 1a. The last case is not possible for binary trees, but it is for trees of arbitrary degrees. The triplet distance is defined as the number of triplets whose topology differs in the two trees. It can naively be computed by enumerating all 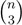 sets of three leafs and for each set comparing the induced topologies in the two trees.

A quartet is a set, {*i*, *j*, *k*, *l*}, of four leaf labels. This is the smallest number of leaves for which the subtree induced by these leaves can have different topologies in two unrooted trees. The possible topologies are shown in Figure 1b. The last case is not possible for binary trees (trees with inner nodes of a degree of three), but it is for trees of arbitrary degrees. For the remaining three cases, the set, {*i*, *j*, *k*, *l*}, is *split* into two sets of two leaves by the middle edge. We sometimes use the notation, *ij*|*kl*, for splits, with this particular instance referring to the leftmost topology in Figure 1b. Similar to the triplet distance, the quartet distance is defined as the number of quartets whose topology differ in the two trees. It can naively be computed by enumerating all 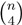 sets of four leafs and for each set comparing the induced topologies in the two trees.

**Figure 2:**
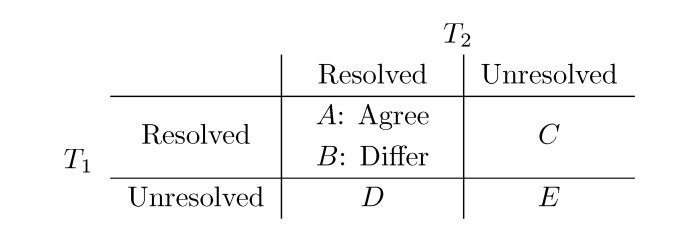
Cases for computing differences.

The rightmost cases for triplets and quartets in Figure 1a,b are called *unresolved*, while the remaining are called *resolved*. Both for triplets and quartets, the possible topologies in two trees, *T*_1_ and *T*_2_, of arbitrary degree can be partitioned into the five cases, *A–E*, listed in Figure 2. Note that in the resolved-resolved case, the triplet/quartet topologies may either agree (*A*) or differ (*B*) in the two trees. In the resolved-unresolved and unresolved-resolved cases (*C* and *D*), they always differ, and in the unresolved-unresolved case (*E*), they always agree. For binary trees, only the two resolved-resolved cases are possible.

Since both distances are defined as the number of differing topologies, they can be computed as *B* + *C* + *D*. However, none of our algorithms compute *B*, *C* and *D* explicitly. Different algorithms use different relationships between the five counters, *A–E*, to compute the distances faster.

The quartet and triplet distances are known to be more robust to small changes in the trees than other distance measures, including the Robinson-Foulds distance [6]. They also have a much larger range than many other distance measures for trees and are, therefore, regarded as more fine-grained. The maximal value of the normalized quartet distance, (*B* + *C* + *D*)/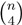, obtained by dividing the distance by the total number of quartets was shown by Bandelt and Dress to be monotonically increasing with *n*, and they conjectured that this ratio is bounded by 2/3 [7]. Steel and Penny furthermore showed that the normalized mean value of the distances, 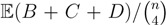, tends to 2/3 as *n* → ∞ and that the variance of (*B* + *C* + *D*)/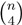 tends to zero as *n* → ∞, when all (binary or general) trees are sampled with equal probability [6]. A further desirable feature of the quartet and triplet distances is that quartets and triplets play a fundamental role in many approaches to phylogenetic tree reconstruction (see, e.g., the R* method in Dendroscope [8] or the Quartet MaxCut method [9]).

The parameterized triplet and quartet distance is defined by Bansal *et al.* [10] as *B* + *p* · (*C* + *D*), for some 0 ≤ *p* ≤ 1, and makes it possible to weigh the contribution of unresolved triplets/quartets to the total triplet/quartet distance. For *p* = 0, unresolved triplets/quartets do not contribute to the distance, *i.e*., unresolved triplets/quartets are ignored, while for *p* = 1, they contribute fully to the distance, as is the case in the unparameterized triplet/quartet distance. Bansal *et al.* recently showed that the parameterized triplet and quartet distances are: i) metrics if *p* ∈ [1/2, 1]; ii) distance measures, but not metrics (the triangle-inequality is violated) if *p* ∈ (0, 1/2); and iii) not distance measures if *p* = 0 (two non-equivalent trees can have a “distance” of zero) [10]. Whether unresolved topologies should contribute to the distance depends on whether one considers an unresolved node a statement of knowledge of a multifurcation or simply a lack of knowledge of the true topology. See, e.g., Pompei *et al.* [11] and Walker *et al.* [12] for a discussion of dealing with unresolved topologies as a lack of knowledge in linguistics.

## 3. Computing the Triplet and Quartet Distances between Binary Trees

In this section, we consider the binary case, *i.e.*, where all topologies are resolved and *C*, *D* and *E* from Figure 2 are zero. In this case, the distances are the *B* counter, which can be obtained by computing either *A* or *B*, since *B* = 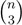 − *A* for the triplet distance and *B* = 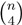 − *A* for the quartet distance.

We will describe two algorithms for computing the quartet distance for binary trees, one based on dynamic programming and the other on coloring leaves and comparing topologies induced by the coloring. These are also the two approaches that we have used to handle general trees, and we will describe the extensions for general trees in the next section. Variations of the two approaches to compute the triplet distance have also been developed, but in our main exposition, we focus on the quartet distance.

### 3.1. A Dynamic Programming Approach

The first algorithm, from Bryant *et al.* [13], is based on two ideas: orienting edges and associating directed edges to quartets and, then, building tables of the number of leaves shared between two subtrees.

**Figure 3:**
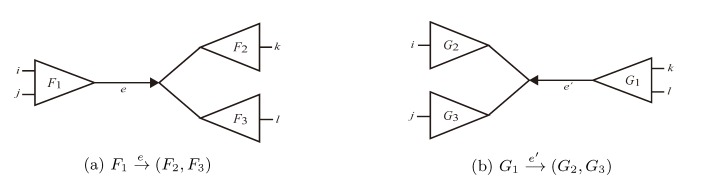
Any quartet is claimed by exactly two edges.

We first conceptually take all edges in the two trees and replace them with two oriented edges. This way, we can uniquely assign three subtrees to each oriented edge: *F*_1_ behind the edge and *F*_2_ and *F*_3_ in front of the edge (see Figure 3a). We will write this as 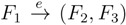 and say that the directed edge, 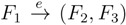, *claims* all quartets, *ij*|*kl*, with *i*, *j* ∈ *F*_1_, *k* ∈ *F*_2_ and *l* ∈ *F*_3_ (or *l* ∈ *F*_2_ and *k* ∈ *F*_3_). If a tree contains the quartet topology, *ij*|*kl*, it will be claimed by exactly two such edges: 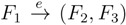 with *i*,*j* ∈ *F*_1_, *k* ∈ *F*_2_ and *l* ∈ *F*_3_ (or *l* ∈ *F*_2_ and *k* ∈ *F*_3_); and 
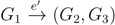
 with *k*, *l* ∈ *G*_1_, *i* ∈ *G*_2_ and *j* ∈ *G*_3_ (or *j* ∈ *G*_2_ and *i* ∈ *G*_3_) as in Figure 3b.

We will consider these two *oriented* quartets, *ij* → *kl* and *ij* ← *kl*, where *e* claims *ij* → *kl* and *e’* claims *ij* ← *kl*. Any shared quartet topology, *ij*|*kl*, corresponds to exactly two oriented quartet topologies, so we can compute *A* by counting the number of shared oriented quartet topologies and divide by two.

Given two trees, *T*_1_ and *T*_2_, the algorithm now simply iterates through all edges, 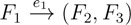 in *T*_1_ and 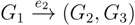 in *T*_2_, and counts how many oriented quartets, *ij* → *kl*, are claimed by both *e*_1_ and *e*_2_. This number, denoted A(*e*_1_,*e*_2_), can be computed as:

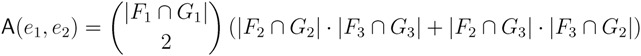

where |*F* ∩ *G*| denotes the number of leaves in two subtrees, *F* and *G*, with the same labels. This expression can be computed in constant time if we have tables containing the count, |*F* ∩ *G*|, for all subtrees, *F* of *T*_1_ and *G* of *T*_2_. The number of shared quartets (and by extension, the quartet distance) can then be computed as:

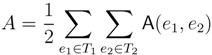

in time *O*(*n*^2^).

Tables for |*F* ∩ *G*| can be computed recursively in time *O*(*n*^2^): if *F* contains subtrees, *F*_1_ and *F*_2_, and *G* contains subtrees, *G*_1_ and *G*_2_, then |*F* ∩ *G*| = |*F*_1_ ∩ *G*_1_| + |*F*_1_ ∩ *G*_2_| + |*F*_2_ ∩ *G*_1_| + |*F*_2_ ∩ *G*_2_|. If *F* or *G* (or both) are leaves, similar, but simpler, cases are used.

By modifying this algorithm slightly, it can also be used to compute the triplet distance between rooted binary trees in *O*(*n*^2^) time.

### 3.2. Tree Coloring

Brodal *et al.*’s *O*(*n* log^2^ *n*) and *O*(*n* log *n*) algorithms [14,15] take a different approach than dynamic programming, but they are also based on oriented quartets. Rather than associating oriented quartets to edges, however, the algorithms associate oriented quartets to inner nodes. Given an inner node, *v*, with three incident subtrees, *F* , *G* and *H*, we say that *v* claims the oriented quartets, *ij* → *kl*, where *i* and *j* are in two different subtrees of *v* and *k* and *l* both are in the remaining subtree. Node *v* thus claims 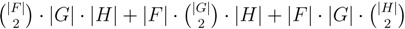 oriented quartets, and similar to before, each quartet is associated with exactly two inner nodes. Furthermore, similar to before, the idea is to compute the double sum 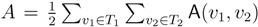, where A(*v*_1_, *v*_2_) now denotes the number of oriented quartets (in the remainder of this section, we will use *quartet* and *oriented quartet* interchangeably) associated with *nodes*, *v*_1_ and *v*_2_, which induce the same quartet topology in both *T*_1_ and *T*_2_. The two algorithms, however, only explicitly iterate over the nodes in *T*_1_, while for each *v*_1_ ∈ *T*_1_, they compute 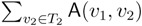 implicitly using a data structure called the *hierarchical decomposition tree* of *T*_2_. To realize this strategy, both algorithms use a coloring procedure in which the leaves of the two trees are colored using the three colors, 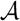, 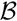 and 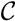. For an internal node, *v*_1_ ∈ *T*_1_, we say that *T*_1_ is *colored according* to *v*_1_ if the leaves in one of the subtrees all have the color, 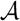, the leaves in another of the subtrees all have the color, 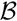, and the leaves in the remaining subtree all have the color, 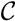. Additionally, we say that a quartet, *ij* → *kl*, is *compatible* with a coloring if *i* and *j* have different colors and *k* and *l* both have the remaining color. From this setup, we immediately get that if *T*_1_ is colored according to a node, *v*_1_ ∈ *T*_1_, then the set of quartets in *T*_1_ that are compatible with this coloring is exactly the set of quartets associated with *v*_1_ and, furthermore, if we color the leaves of *T*_2_ in the same way as in *T*_1_, that the set of quartets, *S*, in *T*_2_ that are compatible with this coloring is exactly the set of quartets that are associated with *v*_1_ *and* induce the same topology in both *T*_1_ and *T*_2_; thus, 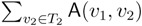 = |*S*|.

Naively coloring the leaves of the two trees according to each inner node in *T*_1_ and counting the compatible quartets in *T*_2_ explicitly would take time *O*(*n*^2^), as we would run through *n* − 2 different colorings and perform *O*(*n*) work for each. By handling the coloring of *T*_1_ recursively and using the “smaller-half trick”, we can ensure that each leaf changes color only *O*(log *n*) times, giving *O*(*n* log *n*) color changes in total. To compute the number of compatible quartets in *T*_2_ for each coloring, we use a hierarchical decomposition of *T*_2_. The main feature of this data structure is that we can decorate it in a way such that it can return the number of quartets in *T*_2_ compatible with the current coloring in constant time. To achieve this, the hierarchical decomposition uses *O*(log *n*) time to update its decoration when a single leaf changes color. Thus, in total, we use *O*(*n* log^2^ *n*) time [14]. The algorithm for computing the triplet distance between binary trees in time *O*(*n* log^2^ *n*) from [16] also uses this strategy, although the decoration of the hierarchical decomposition differs significantly. In the following two subsections, we describe the coloring procedure and the hierarchical decomposition tree data structure used in the quartet distance algorithm. Finally, in Section 3.2.3., we describe how the algorithm was tweaked in [15] to obtain time complexity *O*(*n* log *n*).

#### 3.2.1. Coloring Leaves in *T*_1_ Using the “Smaller-Half Trick”

**Figure 4:**
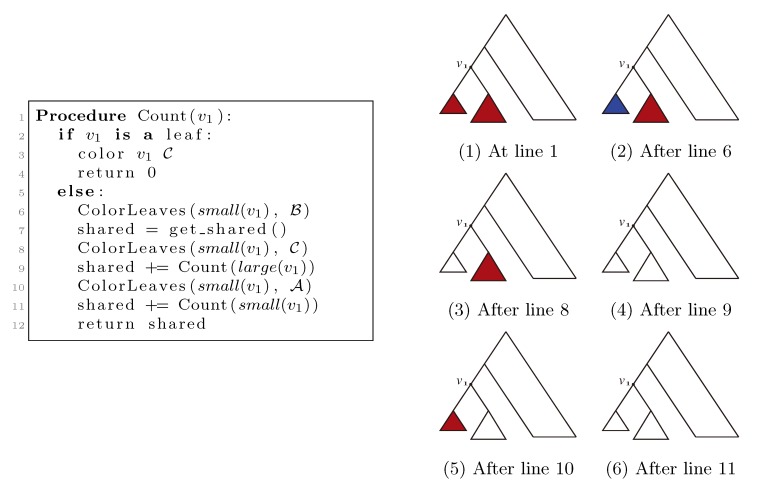
Traversing *T*_1_ using the “smaller-half trick” to ensure that each leaf changes color at most *O*(log *n*) times.

The coloring procedure starts by rooting *T*_1_ in an arbitrary leaf. It then for each inner node, *v*_1_ ∈ *T*_1_, calculates the number of leaves, |*v*_1_|, in the subtree rooted at *v*_1_. This is done in a postorder traversal starting in the new (designated) root of *T*_1_, and the information is stored in the nodes. In this traversal, all leaves are also colored by the color, 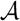, and the new root is colored by the color, 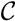. The procedure then runs through all colorings of *T*_1_ recursively, as described and illustrated in Figure 4, starting at the single child of the root of *T*_1_. In Figure 4, large(*v*_1_), respectively small(*v*_1_), denotes the child of *v*_1_ that constitutes the root of the largest, respectively smallest, of the two subtrees under *v*_1_, measured on the number of leaves in the subtrees, and get_shared() is a call to the hierarchical decomposition of *T*_2_ to retrieve the number of quartets in *T*_2_ that are compatible with the current coloring. During the traversal, the following two invariants are maintained for all nodes, *v*_1_ ∈ *T*_1_: (1) when Count(*v*_1_) is called, all leaves in the subtree rooted at *v*_1_ have the color, 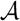, and all other leaves have the color, 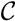; and (2) when Count(*v*_1_) returns, all leaves have the color, 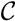. As illustrated in Figure 4, these invariants imply that when get_shared() is called as a subprocedure of Count(*v*_1_), then *T*_1_ is colored according to *v*_1_. Hence, the correctness of the algorithm follows, assuming that get_shared() returns the number of oriented quartets in *T*_2_ compatible with the current coloring of the leaves.

Note that the color of a specific leaf is only changed whenever it is in the smallest subtree of one of its ancestors. Since the size of the smallest of the two subtrees of a node, *v*_1_, is at most half the size of the entire subtree rooted at *v*_1_, this can only happen at most log *n* times for each leaf, leading to *O*(*n* log *n*) color changes in total.

#### 3.2.2. The Hierarchical Decomposition Tree

**Figure 5:**
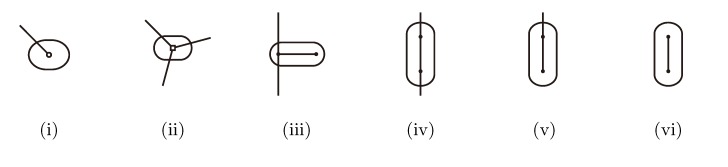
The six different types of components in a hierarchical decomposition tree. Type (**i**)and (**ii**) consist of, respectively, a single leaf or an inner node in the original binary tree. Type (**iii**)–(**vi**) are formed as unions of two adjacent components. Type (**vi**) occurs only at the root of the hierarchical decomposition tree.

The hierarchical decomposition tree of *T*_2_, *HDT*(*T*_2_), is a representation of *T*_2_ as a binary tree data structure with logarithmic height. The nodes in *HDT*(*T*_2_) are called components, and each of them is of one of the six types illustrated in Figure 5. The nodes in *T*_2_ (including leaves) constitute the leaves of *HDT*(*T*_2_): each leaf in *T*_2_ is contained in a component of type (i), and each inner node of *T*_2_ is contained in a component of type (ii). An inner node/component of *HDT*(*T*_2_) represents a connected subset of nodes in *T*_2_, and it is formed as the union of two adjacent components using one of the compositions in Figure 5(iii)–(vi), where two components are adjacent if there is an edge in *T*_2_ connecting the two subsets of nodes they represent. The root of *HDT*(*T*_2_) is formed using the composition in Figure 5(vi), and it represents the entire set of all nodes (including leaves) in *T*_2_.

To build *HDT*(*T*_2_), we first make a component of type (i) or (ii) for each node in *T*_2_ and, then, greedily combine and replace pairs of components using the compositions in Figure 5(iii)–(vi) in subsequent iterations, until we only have the single root component left. This greedy strategy requires *O*(log *n*) iterations using *O*(*n*) time in total, and the result is an HDT of height *O*(log *n*) (see [14,15] for details).

To be able to retrieve the number of quartets in *T*_2_ from *HDT*(*T*_2_) in constant time, we decorate it in the following way: For each component, *C*, in *HDT*(*T*_2_), we store a tuple (*a*, *b*, *c*) of integers and a function, *F*. The integers, *a*, *b* and *c*, denote the number of leaves with colors 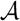, 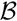 and 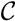, respectively, in the connected component of *T*_2_ represented by *C*. The function *F* takes three parameters for each external edge of *C* (all components have between zero and three external edges, depending on their type, e.g., type (i) components have one external edge, while type (iii) components have two). If *C* has *k* external edges, we number these arbitrarily from one to *k* and denote the three parameters for edge *i* by *a_i_*, *b_i_* and *c_i_*. Of the two ends of an external edge, one is in the component and the other is not. The parameters, *a_i_*, *b_i_* and *c_i_*, denote the number of leaves with colors 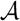, 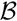 and 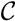, respectively, which are in the subtree of *T*_2_ rooted at the endpoint of edge *i* that is not in *C*. Finally, *F* states, as a function of these parameters, the number of elements of the quartets, which are both associated with nodes contained in *C* *and* compatible with the current coloring of the leaves. It turns out that *F* is a polynomial of a total degree of at most four.

It is beyond the scope of this paper to describe the details of how this decoration of *HDT*(*T*_2_) is initialized and updated, but the crucial point is that for a component, *C*, which is the composition of two components, *C’* and *C”*, the decoration of *C* can be computed in constant time, provided that the decorations of *C’* and *C”* are known. This means that the decoration can be initialized in *O*(*n*) time in a postorder traversal and that it can be updated in *O*(log *n*) time when a single leaf changes color by updating the path from the type (i) component containing the leaf to the root of *HDT*(*T*_2_).

#### 3.2.3. Tweaking the Algorithm to Obtain Time Complexity *O*(*n* log *n*)

To further decrease the time complexity of our algorithm, we need a crucial lemma, which was hinted at in exercise 35 of [17] and stated as Lemma 7 in [15]. This lemma, which was named “the extended smaller-half trick” states that if *T* is a binary tree with *n* leaves and we have a cost of *c_v_* = 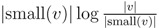 for each inner node *v* and *c_v_* = 0 for each leaf *v*, then we have a total cost of Σ*_v_*_∈_*_T _c_v_* ≤ *n* log *n*. This means that if we can decrease the time used per inner node *v*_1_ in *T*_1_ from |small(*v*_1_)| log *n*, as in the previous two subsections, to 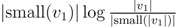, the total time used will be reduced from *O*(*n* log^2^ *n*) to *O*(*n* log *n*).

The first step in doing this is to use Lemma 3 from [15], which tells us that if we are given an unrooted tree, *T* , with *n* nodes of a degree of at most three, where *k* leaves have been marked as non-contractible, then we can contract *T* in *O*(*n*) time into a contraction with at most 4*k* − 5 nodes, such that each non-contractible leaf is a node by itself. This means that if we, prior to visiting a node, *v*_1_ ∈ *T*_1_, mark all leaves in the subtree of *T*_1_ rooted at *v*_1_ as non-contractible and contract *T*_2_ according to this marking, then we get a contraction, *T*_2_^(^*^v^*_1_^)^, with at most 4|*v*_1_|− 5 nodes, and thus, we can build a hierarchical decomposition tree, *HDT*(*T*_2_^(^*^v^*_1_^)^), from this contraction with height *O*(log |*v*_1_|) in time *O*(|*v*_1_|). Hence, if we use *HDT*(*T*_2_^(^*^v^*_1_^)^) instead of *HDT*(*T*_2_) when visiting *v*_1_ ∈ *T*_1_, the time needed for visiting *v*_1_, disregarding the time spent on building *T*_2_^(^*^v^*^)^, is now *O*(|small(*v*_1_)| log|*v*_1_|). However, this is still too much to use the extended smaller-half trick to obtain our goal.

To get all the way down to 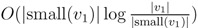, we first observe that a hierarchical decomposition tree is a *locally-balanced* binary rooted tree. A binary rooted tree is *c-locally-balanced* if for all nodes *v* in the tree, the height of the subtree rooted at *v* is at most *c*(1 + log |*v*|). To be exact, our hierarchical decomposition trees are 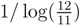-locally-balanced (see [15] for the proof of this). A locally-balanced tree with *n* nodes has the property that the union of *k* different leaf-to-root paths contains just 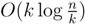 nodes (see Lemma 4 in [15]). Thus, since each *v*_1_ ∈ *T*_1_ *HDT*(*T*_2_^(^*^v^*_1_^)^) has *O*(|*v*_1_|) nodes, the |small(*v*_1_)| leaf-to-root paths in *HDT*(*T*_2_^(^*^v^*^)^) that need to be updated when visiting *v*_1_ contain 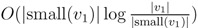 nodes in total. This means that if we spend only constant time to update each of these, we get a total time for coloring and counting, still disregarding the time used to build contractions, of *O*(*n* log *n*) by the extended smaller-half trick. To spend only constant time for each of the 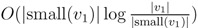 nodes, we split the update procedure for *HDT*(*T*_2_^(^*^v^*_1_^)^) after the |small(*v*_1_)| color changes in two in the following way: (1) We first mark all internal nodes in *HDT*(*T*_2_^(^*^v^*_1_^)^) on paths from the |small(*v*_1_)| type (i) components to the root by marking bottom-up from each of the type (i) components, until we find the first already marked component. (2) We then update all the marked nodes recursively in a postorder traversal starting at the root of *HDT*(*T*_2_^(^^v^_1_^)^).

**Figure 6:**
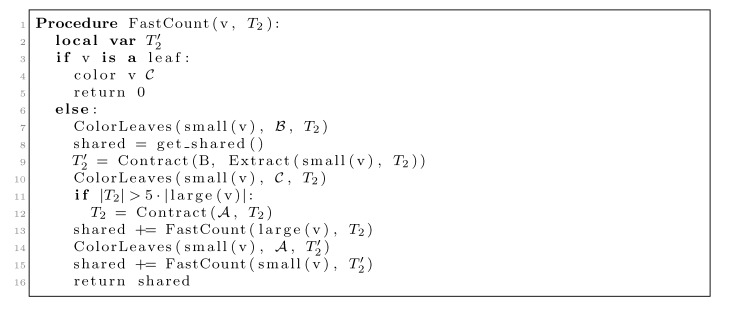
Tree coloring algorithm.

We now use *O*(*n* log *n*) time for the coloring and counting, but if we built *T*_2_^(^^v^_1_^)^ from scratch for every inner node, *v*_1_ in *T*_1_, we would spend *O*(*n*^2^) time just doing this. To fix this problem, we will only contract *T*_2_ whenever a constant fraction of the leaves have been colored 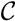, and we will not do it from scratch every time. Figure 6 shows how this is achieved using the subroutines, Contract and Extract. In this pseudo-code, *T*_2_ and $T_{2}^{’}$ are used to refer to the tree, *T*_2_, as well as their associated hierarchical decomposition trees.

The routine, Contract(*X*, *T*_2_), constructs a decomposition of *T*_2_ of size *O*(|*X*|), where each leaf with the color, *X* , has been regarded as non-contractible and where |*X*| is the number of leaves with the color, *X* . This is done in *O*(|*T*_2_|) time, where |*T*_2_| is the number of nodes in the current version of *T*_2_. Note that *T*_2_ is not static, but is a parameter to FastCount and is changed in specific recursive calls. See [15] for the details of Contract. The routine, Extract(small(*v*_1_), *T*_2_), uses the hierarchical decomposition of *T*_2_ to extract a copy of *T*_2_ at the point in the algorithm where *T*_1_ is colored according to *v*_1_. The extracted tree is a copy of *T*_2_ where all leaves in the subtree rooted at small(*v*_1_) still have the color, 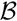, but all other leaves have the color 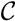. The first call to Contract in line 9 builds a contraction, $T_{2}^{’}$, of this copy, and this contraction is used as *T*_2_ in the recursive call on small(*v*_1_) in line 15. The second call to Contract in line 12 is only executed when |*T*_2_| > 5|large(*v*_1_)| (*i.e*., when more than 4/5 of the leaves have the color, 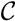). This contraction is used in the recursive call on large(*v*_1_) in line 13.

Line 9 takes 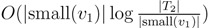 (see [15]); thus, since |*T*_2_| = |*v*_1_| when we perform it, the total time used on this line is *O*(*n* log *n*) by the extended smaller-half trick. We now consider the time spent on contracting *T*_2_ in line 12. We perform this line whenever |*T*_2_| > 5|large(*v*_1_)|. Since all leaves in large(*v*_1_) are colored 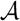 when we contract, the size of the new *T*_2_ has at most 4|large(*v*_1_)| − 5 nodes. Hence, the size of *T*_2_ is reduced by a factor of 4/5. This implies that the sequence of contractions applied to a hierarchical decomposition results in a sequence of data structures of geometrically decaying sizes. Since a contraction takes time *O*(|*T*_2_|), the total time spent on line 12 is linear in the initial size of *T*_2_, *i.e*., it is dominated by the time for constructing the initial hierarchical decomposition tree, which is *O*(*n*). In total, we therefore use *O*(*n* log *n*) time for contracting and extracting *T*_2_.

## 4. Dealing with General Trees

The main focus on algorithms for tree comparison has been on binary trees. This is understandable, since most algorithms for constructing trees will always create fully-resolved trees, even when some edges have very little support from the data (but, see Buneman trees or refined Buneman trees for exceptions to this [18,19]). Trees that are not fully resolved *do* occur in studies, however, and this creates a problem when the algorithms for computing tree distances do not generalize to general trees.

In our research, we have developed a number of approaches for adapting the algorithms from the previous section to work on general trees, and we review these approaches in this section.

### 4.1. Dynamic Programming Approaches

Problems with generalizing the dynamic programming algorithm for binary trees to general trees are two-fold. First, using edges to claim quartets is only meaningful for resolved quartets, and secondly, the resolved quartets claimed by an edge can be distributed over a large number of trees. The first problem can possibly be dealt with by using nodes to claim unresolved quartets, but the second problem is more serious.

We can still compute the tables of the intersections of trees, |*F* ∩ *G*|. If nodes can have a degree up to *d*, then in the recursion for building tables |*F* ∩ *G*|, we need to combine counts for *O*(*d*^2^) pairs of trees, but the total sum of degrees for each tree is bounded by *O*(*n*). Therefore, the product of the pairs of degrees is bounded by *O*(*n*^2^). Worse, however, is computing A(*e*_1_,*e*_2_) where we need to consider all ways of picking trees *F*_2_ and *F*_3_ and pairing them with choices *G*_2_ and *G*_3_, with a worst-case performance of *O*(*d*^4^) for each pair of edges.

In a series of papers, we have developed different algorithms for computing the quartet distance between general trees efficiently by avoiding explicitly having to deal with choosing pairs of trees for inner nodes. Common for these is that we also avoid explicitly handling unresolved quartets, but only consider the resolved topologies and handle the unresolved quartets implicitly.

Christiansen *et al.* [20] developed two algorithms for computing the quartet distance between general trees. The idea in the first algorithm, which runs in time *O*(*n*^3^), is to consider all triplets, {*i*, *j*, *k*}, and count for each fourth leaf-label, *x*, how many of the quartets, {*i*, *j*, *k*, *x*}, have the same topology in the two trees; whether this be resolved topologies, *ix*|*jk*, *ij*|*xk* or *ik*|*jx* counting *A*, or unresolved, (*ijkx*) counting *E*. Counting this way, each shared quartet topology will be counted twelve times; so, the algorithm actually computes 12 · (*A* + *E*), and we must divide by twelve at the end. We explicitly iterate through all *O*(*n*^3^) triplets, and the crux of the algorithm is counting the shared quartet topologies in constant time.

**Figure 7:**
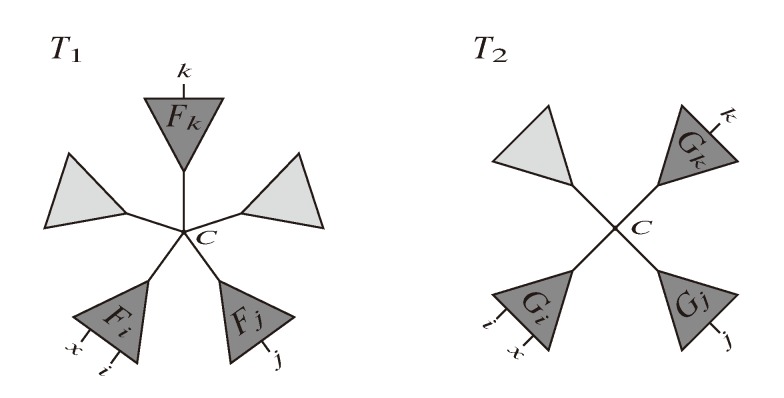
Computing quartets between high-degree nodes.

The approach is as follows: For each triplet, {*i*, *j*, *k*}, we identify the “center”, *C*, of the induced topology in both trees. Let *F_i_*, *F_j_* and *F_k_* be the trees in *T*_1_ connected to *C* containing leaves *i*, *j* and *k*, respectively, and let *G_i_*, *G_j_* and *G_k_* be the corresponding trees in *T*_2_. A resolved quartet, *ix*|*jk*, is shared between the two trees if *x* is in *F_i_* and in *G_i_* (see Figure 7), so the number of such quartets is |*F_i_* ∩ *G_i_*| − 1 (minus one because we otherwise would also count *ii*|*jk*). Therefore, given *i*, *j* and *k*, the number of shared resolved quartets is:

|*F_i_* ∩ *G_i_*| + |*F_j_* ∩ *G_j_*| + |*F_k_* ∩ *G_k_*| − 3

which can be computed in constant time from the tables we build in preprocessing using dynamic programming, assuming we know the centers for the triplet.

Now, let *F_−__ijk_* denote all the leaves in *T*_1_, except those in *F_i_*, *F_j_* and *F_k_*, and similarly, let *G_−__ijk_* denote the set of leaves in *T*_2_ not in *G_i_*, *G_j_* and *G_k_*. The number of unresolved quartets (*ijkx*) is then given by |*F_−__ijk_* ∩ *G_−__ijk_*|. We cannot directly build a table of all |*F_−__ijk_* ∩ *G_−__ijk_*| in *O*(*n*^2^), since just from the number of indices (*i*, *j* and *k*), we would need a table of size *O*(*n*^3^). Instead, we can compute:

|*F_−__ijk_* ∩ *G_−__ijk_*| = |* G_−__ijk_*| − (|*G_−__ijk_* ∩ *F_i_*| + |*G_−__ijk_* ∩ *F_j_*| + |* G_−__ijk_* ∩ *F_k_*|)

where:

|*G_−__ijk_*| = *n* − (|*G_i_*| + |*G_j_*| + |*G_k_*|)

(we can tabulate |*G_l_*| for all *l* in *O*(*n*) in preprocessing) and:

|*G_−__ijk_* ∩ *F_l_*| = |*F_l_*| − (|*F_l_* ∩ *G_i_*| + |*F_l_* ∩ *G_j_*| + |*F_l_* ∩ *G_k_*|)

for *l* = *i*, *j*, *k*; so, we can also count how many unresolved topologies we have for (*ijkx*) if we have built tables in preprocessing and we know the center nodes.

To get the running time of *O*(*n*^3^), we thus only need to find the center nodes in constant time for each triplet, {*i*, *j*, *k*}. We achieve this by finding a linear number of centers, in linear time, for a linear number of triplets. For pairs, {*i*, *j*}, we iterate through all k and their corresponding centers in linear time.

**Figure 8:**
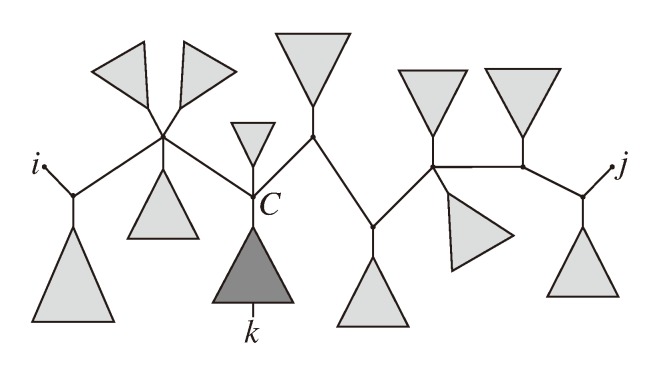
Handling all trees hanging off the path from i to j.

The idea is as follows: for each pair, *i* and *j*, we identify the path between them, which is easily done in *O*(*n*). Along this path, we then consider all inner nodes and the trees branching off. We can explicitly iterate through all *k* in such a subtree, and the corresponding center node is the node where the subtree branches off the path from *i* to *j* (see Figure 8). This way, for each pair, {*i*, *j*}—of which there are *O*(*n*^2^)—we iterate through leaves *k* in *O*(*n*) and count the number of shared quartets, {*i*, *j*, *k*, *x*}, giving us a total running time of *O*(*n*^3^).

The second algorithm in Christiansen *et al.* [20] completely avoids dealing with unresolved quartet topologies by only counting the resolved topologies that are either shared, *A*, or that differ, *B*, between the two trees. Recall that the quartet distance between trees *T*_1_ and *T*_2_ are given by *B*+*C*+*D* in Figure 2. If we have functions A(*T*_1_, *T*_2_) and B(*T*_1_, *T*_2_) that compute *A* and *B* for the two trees, respectively, then we can compute the quartet distance without considering unresolved topologies explicitly, since *A* + *B* + *C* = A(*T*_1_, *T*_1_), *A* + *B* + *D* = A(*T*_2_, *T*_2_), and:

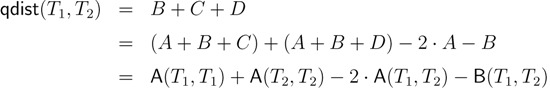

Computing the row and column sums, *A* + *B* + *C* and *A* + *B* + *D*, can also be done faster than using the A function; in fact, it can be computed in linear time for both triplets and quartets using dynamic programming (see either Christiansen *et al.* [21] or Brodal *et al.* [5] for details).

Should one want to compute the parameterized quartet distance instead, it can be done a little more cumbersomely, but still using only the A and B counts:

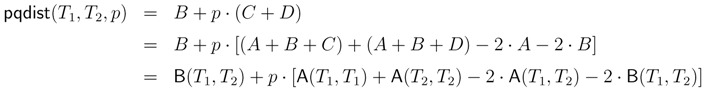

although that is not likely to be an efficient way of doing this. We will not consider it in more detail for now, however.

**Figure 9:**
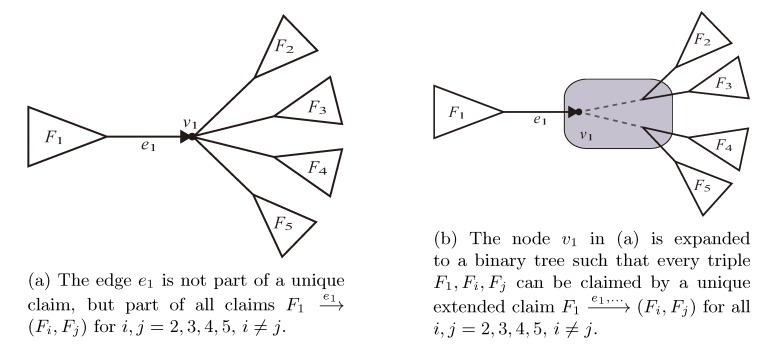
Expanding high-degree nodes.

The second algorithm from Christiansen *et al.* [20] is based on counting the resolved quartets, whether case *A* or *B*, using oriented edges, 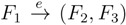, and the quartets they claim. Consider oriented edges *e*_1_ and *e*_2_ in *T*_1_ and *T*_2_, respectively, and let *v*_1_ and *v*_2_ denote the destination nodes of the edges. If *v*_1_ or *v*_2_ are high-degree nodes, then the edges do not correspond to unique claims, 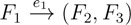 and 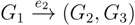, since the trees, *F*_2_, *F*_3_, *G*_2_ and *G*_3_, are not uniquely defined (see Figure 9a). To get around this, the algorithm transforms the trees by expanding (arbitrarily) each high-degree node into a binary tree, tagging edges, so we can distinguish between original edges and edges caused by the expansion (see Figure 9b). We then define *extended claims*, 
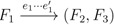
, consisting of two edges, *e*_1_ and $e_{1}^{’}$, such that *e*_1_ is one of the original edges, $e_{1}^{’}$ is a result of expanding nodes and such that the oriented path from *e*_1_ to $e_{1}^{’}$ only goes through expansion edges. The extended claims play the role that claims do in the original algorithm, and each resolved quartet in the original tree is claimed by exactly two extended claims in the expanded tree.

The algorithm then implements functions A and B by explicitly iterating through all pairs of extended claims. Each of the original *O*(*n*) edges are expanded into at most *O*(*nd*) extended claims, where *d* is the maximal degree of the trees, and by counting the equal or different quartet topologies in constant time for each pair of extended claims, the total running time is *O*(*n*^2^*d*^2^).

The A function counts the number of equal topologies from the table of |*F* ∩ *G*| counts exactly as the algorithm for binary trees:

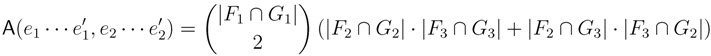

and:

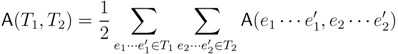



The B function, counting the number of resolved quartets, {*i*, *j*, *k*, *l*}, with different topologies, also uses the tables, but with a slightly different expression. Since we have resolved, but different, quartet topologies whenever 
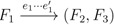
 claims *ij* → *kl* (*i.e*., *i*, *j* ∈ *F*_1_, *k* ∈ *F*_2_ and *l* ∈ *F*_3_) and 
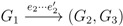
 claims *ik* → *jl*, *il* → *jk*, *jk* → *il* or *jl* → *ik*, we can count as:

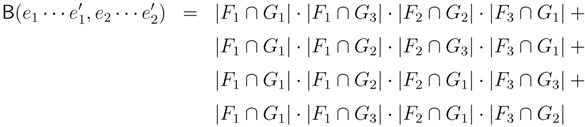



Because of symmetries and counting each quartet in two claims in each tree, this will over-count by a factor of four, so the number of resolved, but different, quartet topologies between the two trees is counted as:

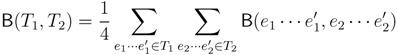



Christiansen *et al.* [21] improved the running time to *O*(*n*^2^*d*) by changing the counting schemes for the A and B functions. The gist of the ideas in the improved counting scheme is to extend the table of shared leaf counts, |*F* ∩ *G*|, with values |*F* ∩ *G*|, |*F* ∩ *G*| and |*F* ∩ *G*|, where *F* denotes the set of leaves *not* in subtree *F*. Using these extra counts, the algorithm builds additional tables, over-counting the number of quartets claimed and, then, adjusting the over-count. For details on this, rather involved, counting scheme, we refer to the original paper [21].

Using the same basic idea, but with yet another counting scheme and another set of tables counting sets of shared leaves, Nielsen *et al.* [22] developed an *O*(*n*^2+^*^α^*) algorithm, where *α* = (*ω* − 1)/2 and *O*(*n^ω^*) is the time it takes to multiply two *n* × *n* matrices. Using the Coppersmith-Winograd algorithm [23], where *ω* = 2.376, this yields a running time of *O*(*n*^2.688^) and was the first guaranteed sub-cubic time algorithm for computing the quartet distance between general trees. Here, the underlying idea is to reduce an explicit iteration over *O*(*d*^2^) pairs of claims to a matrix-matrix multiplication as part of the counting iteration. Again, we refer to the original paper for details [22].

### 4.2. Tree Coloring

The coloring approach of Brodal *et al.* [14,15] for binary trees, described in Section 3.2, has been extended to general trees, first by Stissing *et al.* [24] and recently by Brodal *et al.* [5].

The result of [24] is an *O*(*d*^9^*n* log *n*) time algorithm for computing the quartet distance between two trees, *T*_1_ and *T*_2_, where *d* = max{*d*_1_,*d*_2_} for *d_i_*, the maximal degree of a node in tree *T_i_*. This was the first algorithm for general trees allowing for sub-quadratic time. The approach of [24] stays rather close to that of [14,15], but with a decoration scheme adapted to general trees. The dependency, *d*^9^, on d stems from the HDT data structure having a *d* factor in its balance bound when applied to general trees and from the applied decoration scheme for the HDT requiring *O*(*d*^8^) time for the update of the decoration of a node based on its children after a color change.

The results of [5] are an *O*(*n* log *n*) time algorithm for computing the triplet distance and an *O*(*dn* log *n*) time algorithm for computing the quartet distance, both for general trees, with *d* defined as above. The improved dependency on *d* stems from a new definition of the HDT data structure achieving good balance for general trees and from a much more elaborate decoration scheme, with a large system of auxiliary counters assisting in the calculation of the main counters.

Johansen and Holt [25] have later shown how to improve the algorithm, such that *d* = min{*d*_1_, *d*_2_}, which is of significance if only one of the trees has a high degree.

**Figure 10:**
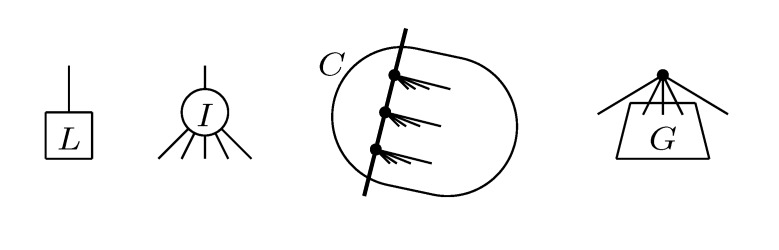
The four different types of components.

**Figure 11:**
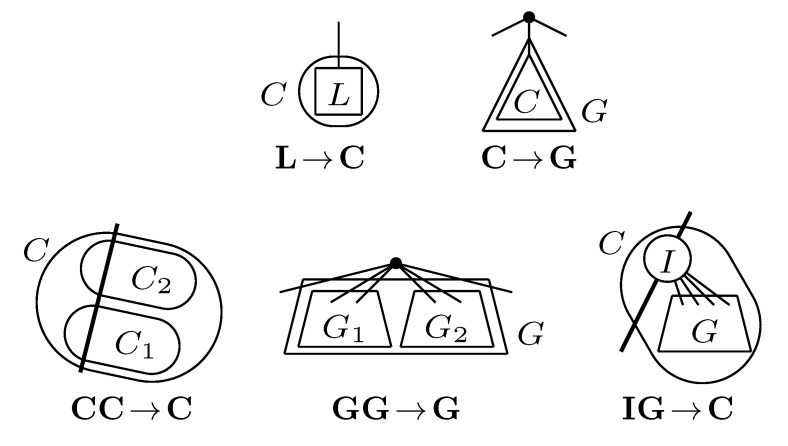
The two types of transformations (**top**) and the three types of compositions (**bottom**).

The new HDT definition is based on the four types of components shown in Figure 10. Here, the base components, constituting the leaves of the HDT, are *L* components containing a leaf from *T*_2_ and *I* components containing a single internal node from *T*_2_. The internal nodes of the HDT are formed by *C* components containing a connected set of nodes with at most two external edges to other components and *G* components containing the subtrees of some of the siblings of a node in *T*_2_. During construction of the HDT, *C* and *G* components are created by the transformations and compositions shown in Figure 11. The construction proceeds in rounds, with each round performing a set of non-overlapping compositions. It is shown in [5] that in *O*(log *n*) rounds and *O*(*n*) time, a 1/ log(10/9)-locally balanced HDT tree is formed. This plays the role of the HDT described in Section 3.2, but now, for general trees.

**Figure 12:**
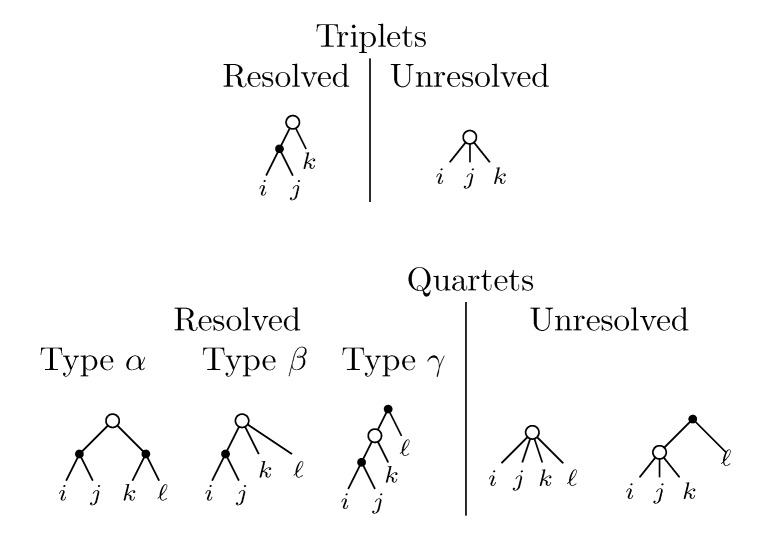
The anchors (white nodes) of resolved and unresolved triplets and quartets. Edges in the figures represents paths in the tree.

Additional changes include the use of *d* + 1 colors, denoted 0, 1, 2,...,*d*, and new definitions of which node a triplet/quartet is associated with. The node in question is denoted the *anchor*, and the choice of anchors can be seen in Figure 12. This choice forms the basis for the definition of the decoration system, which, as said, is quite involved and for which we refer to [5] for details (as well as to [25] for minor corrections and for a trimmed variant of the system).

**Figure 13:**
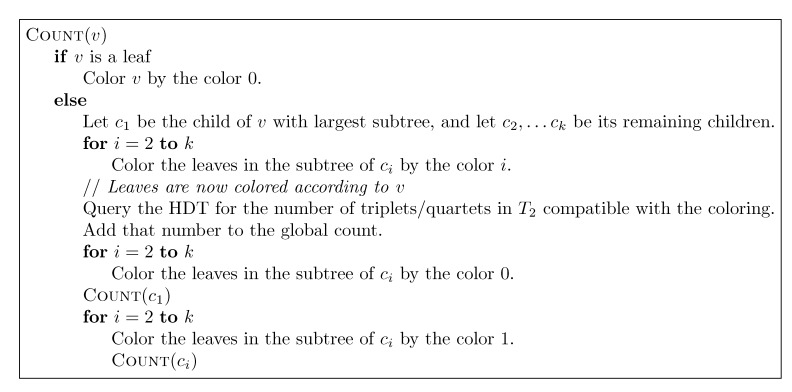
The main algorithm performing a recursive traversal of *T*_1_.

The remaining parts follow the lines of [14,15] (see Section 3.2). The basic algorithm, corresponding to Figure 4 for binary trees, for traversing *T*_1_ recursively can be seen in Figure 13. It maintains the following invariants: (1) When entering a node, *v*, all leaves in the subtree of *v* have the color, one, and all leaves not in the subtree of *v* have the color, zero; (2) When exiting *v*, all leaves in *T*_1_ have the color, zero. To initialize Invariant (1), all leaves are colored one at the start. As in Section 3.2, the operations, Contract and Extract, are then added (analogously to Figure 6) for exploiting a variant of the “extended smaller-half trick”, in order to arrive at the stated running times.

## 5. Experimental Results

Most of the algorithms we have described in this review paper have been implemented in different software tools, and in this section, we experimentally compare their runtime performance. All experiments involved compare randomly generated balanced trees. We note that two randomly generated trees are expected to have a large distance, which influences the running time for the coloring algorithms. Similar trees require, overall, less updating in the hierarchical decomposition, so random trees are a worst-case situation for these. Experiments, not shown here, demonstrate that comparing similar trees can be significantly faster [25]. The shape of the trees also affects the running time for the coloring algorithms through the “smaller-half trick”, with faster running times for very skewed trees (for perfectly balanced trees, the number of color changes required is *Θ*(*n* log *n*), while for the other extreme, caterpillar trees, the number is *Θ*(*n*)).

All experiments in this section were conducted on an Ubuntu Linux Server 12.04, 3.4 GHz 64-bit Intel Core i7-3770 (quad-core) with 32 GB of RAM.

For the quartet distance between binary trees, we performed experiments with three algorithms, the *O*(*n* log^2^ *n*) time algorithm implemented in QDist [14,26], the *O*(*n*^2.688^) time algorithms from Nielsen *et al.* [22] and the *O*(*dn* log *n*) time algorithm from Brodal *et al.* [5]. For the quartet distance between non-binary trees, we considered trees with a degree of eight and 128, using only the last two of the three algorithms, since the QDist algorithm only works on binary trees. Note, however, that the implementation from [5] is not strictly the variation described in the paper. It contains algorithmic optimizations improving both the asymptotic and practical runtime as described in [25].

**Figure 14:**
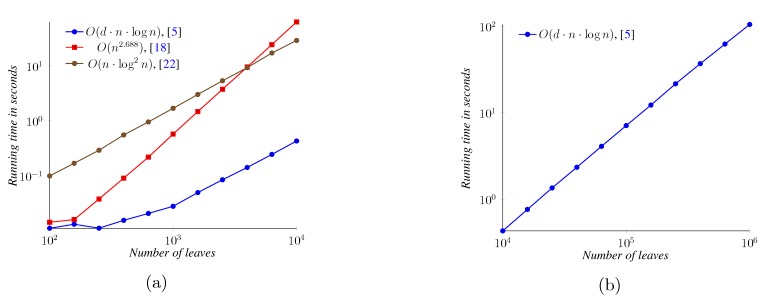
Quartet distance running time on binary trees.

Figure 14 shows the runtimes of the three implementations of the quartet distance calculation algorithms running on binary trees. All three implementations can operate on trees of a size of up to 10,000; only the algorithm from [5] is shown for up to one million leaves. For inputs of less than approximately 4,000 leaves, the implementation of the *O*(*n*^2.688^) time algorithm is faster than the implementation of the *O*(*n* log^2^ *n*) time algorithm. For all input sizes depicted, however, the implementation of the *O*(*dn* log *n*) time algorithm is the fastest, computing the distance between two trees with one million leaves in well under two minutes.

**Figure 15:**
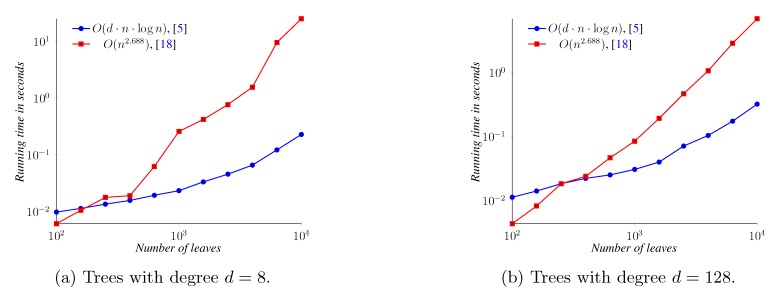
Quartet distance running time on non-binary balanced trees.

Figure 15a compares the two general quartet distance algorithms on trees with a degree of eight, while Figure 15b compares the algorithms for trees with a degree of 128. Both algorithms are faster on these higher-degree trees than on binary trees. The *O*(*n*^2.688^) time algorithm is faster on small trees (where the exact point where the other algorithm is faster depends on the degree).

We note, however, that the runtime of the algorithm from [5], for *d* somewhere between 256 and 512, in fact, increases beyond that of the binary case. For *d* = 1,024, the runtime of the algorithm has almost doubled compared to the binary case, and for *d* = 2,048, the runtime has more than tripled compared to the binary case (results not shown).

**Figure 16:**
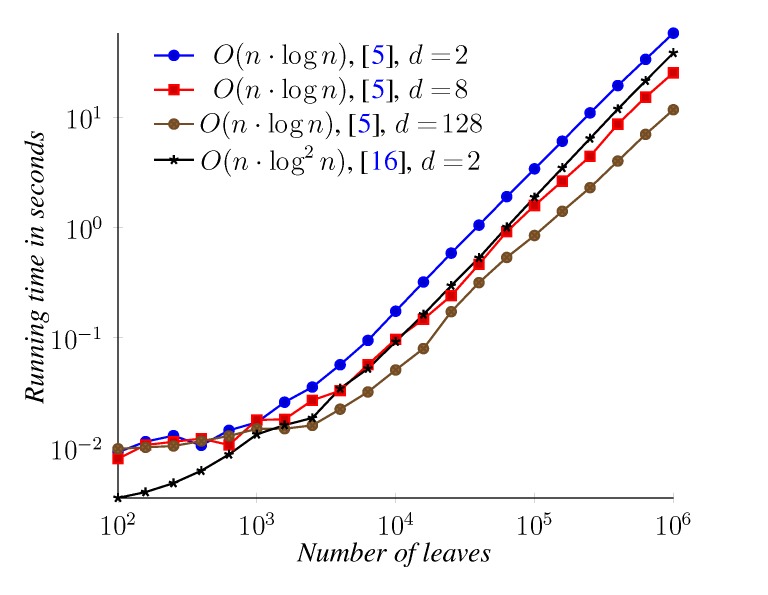
Triplet distance running time. For the *O*(*n* log^2^ *n*) time algorithm, which only handles binary trees, results are only shown for a degree of *d* = 2, while for the general algorithm, results are also shown for *d* = 8 and *d* = 128.

For the triplet distance between binary trees, we compared the general algorithm from [5] with the algorithm from [16] for binary trees. For the general algorithm, we also performed experiments on trees with a degree of 8 and 128. Figure 16 shows the measured running times. These experiments are summarized in Figure 16. For binary trees, the *O*(*n* log^2^ *n*) time algorithm [16] is faster than the *O*(*n* log *n*) time algorithm [5] for all measured sizes. For the general algorithm, we again observe that it performs faster on high-degree trees (where, since the number of leaves is fixed, we have fewer internal nodes to handle).

## 6. Conclusions

We have presented a series of algorithmic improvements for computing the triplet and quartet distance between two general trees that we have developed over the last decade. Our work has followed two main approaches, one based on counting shared topologies using tables of the intersections of subtrees and one based on coloring labels and counting compatible topologies using a hierarchical decomposition data structure. The second approach has resulted in the currently best worst-case running time of *O*(*n* log *n*) for computing the triplet distance and *O*(*dn* log *n*) for computing the quartet distance. Whether this can be improved further is currently unknown.

While the theoretical fastest algorithms involve rather complex bookkeeping for counting topologies, we have shown that they can be implemented to be efficient in practice, as well, computing the distance between two trees with a million leaves in a few minutes. With more typical phylogenetic tree sizes, with the number of leaves in the hundreds or low thousands, the distance can be computed in less than a second.
